# Examining the moderating role of technostress and compatibility in EFL Learners’ mobile learning adoption: A perspective from the theory of planned behaviour

**DOI:** 10.3389/fpsyg.2022.919971

**Published:** 2022-08-10

**Authors:** Qiong Wang, Guoqing Zhao, Zhuo Cheng

**Affiliations:** ^1^College of Science (Teachers College), Shaoyang University, Shaoyang, China; ^2^School of Educational Technology, Faculty of Education, Beijing Normal University, Beijing, China

**Keywords:** technostress, theory of planned behaviour (TPB), compatibility, mobile English learning, undergraduates

## Abstract

Mobile English learning has multiple advantages and brings enormous benefits to EFL learners. However, not everyone adopts it, and the determinants of learners’ adoption intention have not yet been investigated fully. This study aimed to better understand learners’ adoption by employing the theory of planned behaviour (TPB) in conjunction with the influences of technostress and compatibility. Based on existing literature, a research model was proposed and verified with a sample of 409 undergraduates from a Chinese university. The results indicated that in the context of mobile English learning: (a) Individuals with higher adoption intention are more likely to engage in mobile English learning with higher frequency (*b* = 0.473, *P* < 0.001) and longer duration (*b* = 0.330, *P* < 0.001); (*b*) Individuals’ attitude toward mobile English learning (*b* = 0.171, *P* < 0.05), perceived behavioural control (*b* = 0.221, *P* < 0.001), subjective norms (*b* = 0.237, *P* < 0.05), and compatibility (*b* = 0.443, *P* < 0.001) are significantly positively associated with their adoption intention; (c) Compatibility is the strongest predictor of adoption intention (*b* = 0.443, *P* < 0.001) and negatively moderates the effect of subjective norms on adoption intention (*b* = –0.103, *P* < 0.005); (d) The influence of technostress on the adoption intention of mobile English learning is not significant (*b* = –0.041, *P* > 0.05). Practical implications related to mobile English learning were discussed.

## Introduction

Mobile English learning refers to learning English through portable mobile devices (e.g., smartphones, tablets, iPod/iPod touch; Chen et al., 2019). It embraces various advantages, including multimedia content, portability, the flexibility of space and time, and has been widely used by EFL learners (Lee and Sylvén, 2021). A number of English learning apps or platforms have been developed, some of which have received a number of users. For instance, ‘‘Baicizhan,’’ a well-known English vocabulary learning app in China, has more than one million active users daily.^1^ However, not everyone wants to apply mobile technologies for learning (Wang et al., 2009; Park, 2011). Some individuals do not intend to use or are unwilling to continue using mobile devices for English learning (Xi et al., 2020). The advantages of mobile English learning cannot guarantee learners’ adoption. Investigation the mechanism of EFL learners’ adoption intention of mobile English learning can provide targeted evidence for developers of apps and platforms.

The theory of planned behaviour (TPB) is an inspirational theory to explain human behaviour (Ajzen, 1991). According to the TPB, attitude toward behaviour, perceived behavioural control, and subjective norms are three key determinants of behavioural intention. Researchers employed the TPB to explore the antecedents of mobile learning adoption and found that TPB has good explanatory power in predicting users’ adoption intention of mobile learning (Cheon et al., 2012; Chu and Chen, 2016). In addition, Nie et al. (2020) utilised the TPB to explore learners’ adoption of mobile English learning check-in behaviour in China.

However, the existing studies have been limited at least in these aspects: (1) The results are conflicting. For instance, some studies suggested a significant and positive association between subjective norms and technology adoption intention (Sentosa and Mat, 2012; Sawang et al., 2014; Chu and Chen, 2016), while some other researchers reported no significant association (Yuen and Ma, 2008; Knauder and Koschmieder, 2019; Nie et al., 2020). (2) Research employing the TPB to examine the antecedents of EFL learners’ mobile English learning is scarce. (3) They did not consider the impacts of other variables viewed as essential predictors of technology adoption, such as technostress and compatibility (Ragu-Nathan et al., 2008; Jaklič et al., 2018). Technostress refers to users’ stress due to their disability to deal with the technology demands in working and learning (Maier et al., 2019). Compatibility refers to the matching degree between IT innovation and its potential adopters’ needs (Jaklič et al., 2018).

Therefore, this study strived to understand the adoption of EFL learners’ mobile learning employing the TPB combined with the effects of technostress and compatibility. Specifically, the research questions are as follows:

RQ1: Does the TPB have good explanatory power in predicting EFL learners’ mobile learning behaviour?

RQ2: How technostress influences EFL learners’ mobile learning behaviour?

RQ3: How compatibility influences EFL learners’ mobile learning behaviour?

## Literature review and hypotheses development

### The theory of planned behaviour

**FIGURE 1:**
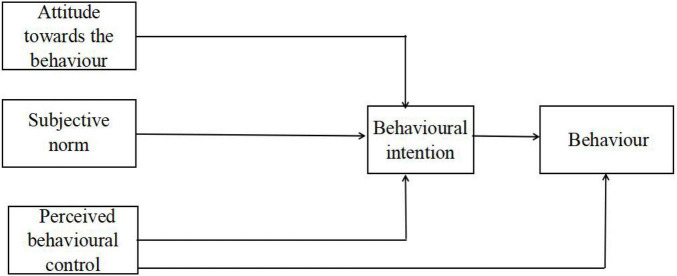
The theory of planned behaviour.

The theory of planned behaviour (TPB) was developed to explain the behaviours of human beings, which has been confirmed empirically to predict behaviours in a variety of settings (Ajzen, 1991; Ozkan and Kanat, 2011). According to the TPB, an individual’s attitude toward behaviour, perceived behavioural control, and subjective norms are three key antecedents of behavioural intention (Figure 1). Many researchers employed the TPB to understand users’ technology adoption intention (Ozkan and Kanat, 2011; Chu and Chen, 2016; Luqman et al., 2018; Nie et al., 2020). For instance, Chu and Chen (2016) utilised the TPB combined with group influences to examine the antecedents of people’s intention of e-learning adoption. Their findings are also consistent with the assumption of the TPB. Since existing research has confirmed TPB’s explanatory power in predicting individuals’ technology adoption, it is plausible to expect that the TPB can explain users’ adoption intention of mobile English learning.

### Behavioural intention and behaviour

Behavioural intention is the cognitive representation of individuals’ willingness to participate in specific behaviour and is viewed as the forerunner of behaviour (Ajzen, 1991). Many studies suggested that technology adoption intention positively predicts technology usage behaviour (Merhi, 2015; Nie et al., 2020). In the context of mobile English learning, we also expect that users’ intention positively predicts their behaviour. Therefore, the present study proposed the following hypothesis:

H1: Users’ mobile English learning adoption intention is positively associated with their mobile English learning behaviour.

Referring to the research of Nie et al. (2020), the present study identified two indicators (i.e., learning frequency and duration) to examine users’ mobile English learning behaviour. Thus, H1 included the following two sub-hypotheses:

H1a: Users with higher intention would use mobile English learning more frequently.

H1b: Users with higher intention would insist on using mobile English learning for a longer time.

### Antecedents of behavioural intention

#### The attitude toward the behaviour

The attitude toward the behaviour is defined as “the degree to which a person has a favourable or unfavourable evaluation of the behaviour in question” and is considered a critical factor affecting an individual’s behavioural intention (Ajzen, 1985). The attitude toward the behaviour includes people’s appraisal of their preferences for specific behaviour and their judgement of the possible consequences of participating in the behaviour. Many empirical studies have found that people’s attitude toward the behaviour is positively associated with their behavioural intention (Faham and Asghari, 2019; Chen and Wu, 2020). In terms of technology adoption, researchers have also found that individuals’ attitude toward technology is an important precursor (Avidov-Ungar and Eshet-Alkalai, 2011; Hoi, 2020; Nie et al., 2020). For instance, Avidov-Ungar and Eshet-Alkalai (2011) found that teachers’ attitude toward innovative technology is a key predictor of their technology adoption. Therefore, the present study proposed the following hypothesis:

H2: Users’ attitude toward mobile English learning is positively associated with mobile English learning adoption intention.

#### Perceived behavioural control

Perceived behavioural control refers to individuals’ judgement on their resources and capabilities to participate in a given behaviour (Ajzen, 1985). It concerns the influences of internal and external factors on behaviour. Individuals with a higher perception of resources and ability related to behaviour are more likely to engage (Cheon et al., 2012). Perceived behavioural control has been proved to be significantly correlated with behavioural intention in different settings, such as learning, consumption and government services (Ozkan and Kanat, 2011; Chu and Chen, 2016; Nie et al., 2020). Specifically, perceived behavioural control was a positive predictor of individuals’ e-learning adoption, mobile shopping adoption, and intention to use e-government services. In the context of mobile English learning, individuals’ perceived behavioural control may also positively predict their adoption intention. Therefore, the present study formulated the following hypothesis:

H3: Users’ perceived behavioural control is positively associated with their mobile English learning adoption intention.

#### Subject norms

Subjective norms refer to “the perceived social pressure to perform or not to perform the behaviour” and are critical determinants of human behaviours (Ajzen, 1985). They denote the impact of people (e.g., parents, friends, and colleagues) who are very important to individuals on their participation in a specific behaviour. However, existing empirical studies about the influence of subjective norms on behavioural intention are inconsistent. Some studies suggested a significant and positive association between subjective norms and behavioural intention (Sentosa and Mat, 2012; Sawang et al., 2014; Chu and Chen, 2016). However, other researchers found no significant association (Yuen and Ma, 2008; Knauder and Koschmieder, 2019; Nie et al., 2020). Additionally, some researchers added boundary conditions to understand the effects of subjective norms on behavioural intention better. For instance, Chu and Chen (2016) proposed social identity and social bond as moderators. To further explore the relationship between subjective norms and behavioural intentions in the context of mobile English learning, this study temporarily posited that subjective norms positively predict behavioural intention based on the TPB and proposed two moderating variables (i.e., compatibility and technostress) in this association. Therefore, the present study proposed the following hypothesis:

H4: Subjective norms are positively associated with mobile English learning adoption intention.

#### Technostress

Technostress, a psychological strain caused by the use of technologies, is defined as individuals’ affective and cognitive stress triggered by technical demands of work (Tarafdar et al., 2019). It is an unintended consequence of the application of technologies for multiple purposes. With the broad penetration of information technologies into education, students may face increasing technostress (Lee et al., 2014; Boonjing and Chanvarasuth, 2017; Yao and Cao, 2017). Additionally, further studies suggested that technostress has a significant and negative influence on students’ academic performance and productivity and is positively associated with learning burnout (Qi, 2019; Upadhyaya and Vrinda, 2021; Zhao et al., 2022). Researchers have also examined the impact of technostress on individuals’ technology adoption (Joo et al., 2016; Luqman et al., 2018; Chen et al., 2019; Verkijika, 2019). For instance, Joo et al. (2016) found that technostress is negatively associated with teachers’ intention to use technology. Additionally, Verkijika (2019) found that technostress plays a negative moderator in the relationship between perceived usefulness and adoption intentions of digital textbooks, suggesting that the impact of perceived usefulness on adoption intention is weaker when technostress is higher. Steelman and Soror (2017) suggested that individual’s cognitive evaluation about a given technology may be affected by negative psychological states such as technostress. Since individuals’ technostress may vary due to their characteristics (Marchiori et al., 2019), technostress may be a boundary condition to explain the conflicting results about the relationship between subjective norms and behavioural intention. Thus, this study proposed that technostress may negatively predict learners’ intention of participating in mobile English learning and play a possible moderator in the influence of subjective norms on behavioural intention. Therefore, we formulated the following hypotheses:

H5a: Technostress is negatively associated with mobile English learning adoption intention.

H5b: Technostress moderates the effect of subjective norms on mobile English learning adoption intention.

#### Compatibility

Compatibility refers to the degree to which the innovation is viewed in line with its potential users’ existing values, past experiences, and current needs (Moore and Benbasat, 1991). In this study, we broadly defined compatibility in the context of mobile English learning as the degree to which mobile learning matches EFL learners’ learning preferences and experiences. According to Rogers’ innovation diffusion theory, compatibility is a key antecedent of users’ technology acceptance (Rogers, 1983; Kaur Kapoor et al., 2014). Researchers from management, computer science, and psychology also have confirmed that compatibility is an important predictor of individuals’ behavioural intention (Kim et al., 2010; Bulent et al., 2016). For instance, Shin et al. (2018) confirmed that compatibility has an important influence on smart home technology acceptance. Therefore, it is reasonable to posit that compatibility may be a positive predictor of EFL learners’ adoption of mobile English learning. However, compatibility is not only a predictor of users’ behaviour intention, but also closely linked with users’ attitudes (Ho et al., 2020; Tsai and Tiwasing, 2021). Additionally, Wang et al. (2020) found that compatibility positively moderates the relationship between perceived value and usage intention in the m-government context. Considering mobile English learning has both advantages and disadvantages, whether it compatible with EFL learners’ learning preferences and needs may affect not only their intentions to use, but also may have a moderating effect on the relationship between independent variables and intention to use. As previous findings about the influence of subjective norms on behavioural intention are inconsistent, this study suggested that compatibility may play a moderator in the relationship between subjective norms and behavioural intention. Therefore, the present study proposed the following hypotheses:

H6a: Compatibility is positively associated with mobile English learning adoption intention.

H6b: Compatibility moderates the effect of subjective norms on mobile English learning adoption intention.

### The present study

**FIGURE 2:**
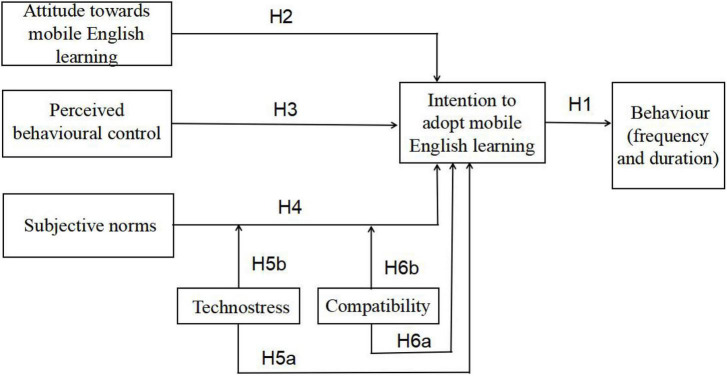
The research model.

This study aimed to explore the impact of EFL learners’ attitude toward mobile learning, perceived behavioural control, and subjective norms on their intention to adopt mobile English learning by employing the TPB and examining the moderating role of technostress and compatibility in the relationship between subjective norms and adoption intention. The proposed research model was shown in Figure 2, in which attitude, perceived behavioural control, subjective norms, adoption intention, technostress, and compatibility are latent variables; behaviour is an observable variable with two indicators (i.e., frequency and duration).

## Materials and methods

### Participants

The survey was performed in April 2021 (from April 15 to April 30). A convenience sampling approach was used to collect data. A public university in southern China was selected, where learning English with mobile devices is very popular among students. This study developed an online questionnaire and sent it to students to invite them to participate voluntarily and anonymously. The online survey link was sent to students during classroom meetings by the student counsellors, who briefly introduced the purpose of this study to ensure that participants were familiar with or had experience of mobile English learning. The survey was anonymous and voluntary. A total of 409 valid responses were received. Among them, 84.84% had more than 1 h of mobile English learning per week, while 75.55% had mobile English learning experience more than once a week. The demographic information and the mobile English learning experiences of the participants were shown in Table 1.

**TABLE 1:** Demographic information of the participants.

Gender	Number	Percentage	Duration (hours per week)	Number	Percentage
Female	248	60.64%	<1 (much shorter)	62	15.16%
Male	161	39.36%	1–3 (shorter)	199	48.66%
*Age*			3–5 (about the same)	68	16.63%
<20	274	66.99%	5–7 (longer)	41	10.02%
21∼23	132	32.27%	>7 (much longer)	39	9.54%
>24	3	0.73%	*Frequency* (times per week)		
*Learning devices*			<1 (very rarely)	100	24.45%
Smartphone	352	86.06%	1–3 (rarely)	175	42.79%
Tablet	4	0.98%	3–6 (sometimes)	80	19.56%
Smartphone and tablet	53	12.96%	6–12 (very often)	30	7.33%
*Learning content* (multiple choices)			>12 (always)	24	5.87%
Vocabulary	371	90.71%			
Listening	231	56.48%			
Speaking	102	24.94%			
Reading	106	25.92%			
Others	82	20.05%			

### Instruments development

This study developed a structured survey including two parts. In the first part, questions were designed to obtain respondents’ demographic information and their experiences of mobile English learning, including their learning frequency (from very rarely to always) and learning duration (from much shorter to much longer) of mobile English learning. Specifically, the following two items were asked to evaluate EFL learners’ mobile English learning behaviour: “How long is your average duration of using mobile English learning per week?” and “How often do you use mobile English learning per week?”

In the second part, items were designed to obtain empirical data to examine the six latent variables in the research model. The items were all adopted and adapted from the previous literature with satisfactory reliability and validity. There were 20 items in total, presented by a 5-point Likert scale with options from “strongly disagree” (1) to “strongly agree” (5).

Specifically, the subscales to examine the four constructs of TPB (i.e., attitude, perceived behavioural control, subjective norms, and behavioural intention) were adopted from Cheon et al. (2012) and Chu and Chen (2016). Each construct was examined by three items. The subscale of technostress was revised from Yao and Cao (2017). In total, there were five items. The compatibility subscale was designed with reference to Moore and Benbasat (1991). In total, there were three items.

### Data analysis

Data were analysed using Mplus (version 7.4). First, confirmatory factor analysis (CFA) was conducted to evaluate the reliability and validity of the measurement model. Second, a structural equation model (SEM) was conducted to test the proposed hypotheses. Third, the moderating effects of technostress and compatibility were calculated. Unstandardized coefficients were reported.

## Results

### The measurement model

The reliability was examined by the factor loadings of items and the Cronbach’s alpha coefficient of constructs. The results of CFA (shown in Table 2) indicated that, excepting CM3 and TS2, the factor loadings of all items are above 0.70, ranging from 0.721 to 0.939. Hence, CM3 and TS2 were removed (Schumacker and Lomax, 2004). In addition, the Cronbach’s alpha coefficients of the six constructs are all above 0.70, ranging from 0.752 to 0.946, suggesting satisfactory reliability.

**TABLE 2:** Results of construct validity and reliability analysis.

Latent variable	Measurement variable	Mean	Std. Dev.	Factor loadings	α
ATT	ATT1	3.94	0.822	0.791	0.904
	ATT2			0.900	
	ATT3			0.939	
PBC	PBC1	3.94	0.786	0.781	0.752
	PBC2			0.897	
	PBC3			0.847	
SN	SN1	3.91	0.791	0.895	0.882
	SN2			0.848	
	SN3			0.796	
TS	TS1	2.89	0.938	0.798	0.888
	TS3			0.892	
	TS4			0.721	
	TS5			0.869	
CM	CM1	3.80	0.849	0.964	0.946
	CM2			0.930	
IN	IN1	3.97	0.820	0.923	0.933
	IN2			0.934	
	IN3			0.872	

ATT, attitude; PBC, perceived behavioural control; SN, subjective norms; TS, technostress; CM, compatibility; IN, intention.

Excepting for the construct of compatibility, the coefficients of composite reliability (CR) and average variance extracted (AVE) of the remaining five constructs were calculated to examine the discriminant and convergent validities. The calculation results (shown in Table 3) revealed that the coefficients of CR are above 0.70 (ranging from 0.880 to 0.935), and coefficients of AVE are above 0.50 (ranging from 0.677 to 0.828). Additionally, the square root of each construct’s AVE is greater than the construct’s correlation coefficients with other constructs. These results suggested that the discriminant and convergent validities of constructs are acceptable (Fornell and Larcker, 1981). Furthermore, the fitness of the measurement model (χ2 = 323.274, df = 120, χ2/df = 2.694, TLI = 0.963, CFI = 0.971, SRMR = 0.034, RMSEA = 0.064) was satisfactory according to the criteria proposed by Hair et al. (2014).

**TABLE 3:** The results of discriminant and convergent validities.

Constructs	CR	AVE	1	2	3	4	5	6
ATT	0.910	0.773	0.879					
PBC	0.880	0.711	0.698	0.843				
SN	0.884	0.718	0.824	0.719	0.847			
TS	0.893	0.677	0.082	0.054	0.100	0.823		
CM	—	—	0.713	0.676	0.736	0.116	—	
IN	0.935	0.828	0.791	0.751	0.805	0.007	0.841	0.910

CR, composite reliability; AVE, average variance extracted; ATT, attitude; PBC, perceived behavioural control; SN, subjective norms; TS, technostress; CM, compatibility; IN, intention.

Since the construct of compatibility had only two valid items, its CR and AVE were unavailable. However, a two-item scale is also acceptable with a good Cronbach’s alpha coefficient (Eisinga et al., 2013). In this study, the Cronbach’s alpha coefficient of compatibility (0.946) was ideal, and the other values of the measurement model were all satisfactory. Therefore, we can conclude that the reliability and validity of the measurement model were acceptable.

### The structural model

**FIGURE 3:**
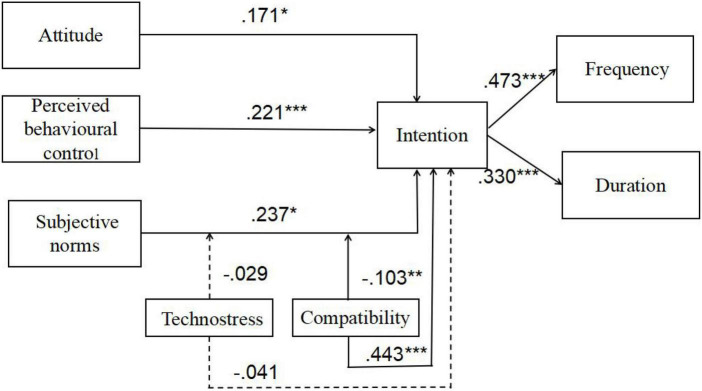
The research model with its path coefficients. ***p < 0.001, **p < 0.01, *p < 0.05.

Hypotheses testing results (shown in Table 4) indicated that EFL learners’ adoption intention significantly predicted both mobile English learning frequency (*b* = 0.473, *P* < 0.001) and duration (*b* = 0.330, *P* < 0.001), supporting H1a and H1b. Learners’ attitude toward mobile English learning (*b* = 0.171, *P* < 0.05), perceived behavioural control (*b* = 0.221, *P* < 0.001), and subjective norms (*b* = 0.237, *P* < 0.05) were all positively associated with their adoption intention, supporting H2, H3, and H4. However, technostress had no significant effect on behavioural intention (*b* = –0.041, *P* > 0.05), and its moderating effect was not significant (*b* = –0.029, *P* > 0.05), rejecting H5a and H5b. Compatibility positively predicted behavioural intention (*b* = 0.443, *P* < 0.001) and played a negative moderator in the effect of subjective norms on intention (*b* = –0.103, *P* < 0.005), supporting H6a and H6b. The research model with its path coefficients is shown in Figure 3.

**TABLE 4:** The results of hypotheses testing.

Hypotheses	Hypothesised path	*b*	S.E.	*t*	Result
H1a	IN → Frequency	0.473	0.068	6.949***	Supported
H1b	IN → Duration	0.330	0.066	5.019***	Supported
H2	ATT → IN	0.171	0.086	1.994*	Supported
H3	PBC→IN	0.221	0.06	3.710***	Supported
H4	SN →IN	0.237	0.1	2.363*	Supported
H5a	TS → IN	–0.041	0.022	–1.855	Rejected
H5b	Moderating effects of TS	–0.029	0.046	–0.629	Rejected
H6a	CM → IN	0.443	0.045	9.874***	Supported
H6b	Moderating effects of CM	–0.103	0.03	–3.369**	Supported

b, unstandardized coefficients; IN, intention; ATT, attitude; PBC, perceived behavioural control; SN, subjective norms; TS, technostress; CM, compatibility.

***p < 0.001, **p < 0.01, *p < 0.05.

## Discussion

The current study investigated the determinants of EFL learners’ adoption of mobile English learning employing the TPB in conjunction with the effects of technostress and compatibility. To this end, the direct influences of attitude, subjective norms, perceived behavioural control, technostress, and compatibility on intention were examined. The relationships between adoption intention and learning behaviour were tested. The moderating effects of technostress and compatibility on subjective norms on intention were also examined.

### TPB explains the adoption of mobile English learning well

For one thing, the findings indicated that EFL learners’ adoption intention is significantly and positively associated with their mobile English learning behaviour, which is in line with TPB and the existing literature that have reported the positive association between intention on behaviour (Ajzen, 1985; Lee et al., 2014; Nie et al., 2020). Individuals with higher adoption intention are more likely to engage in mobile English learning with higher frequency and longer duration. For another, the results indicated that attitude, perceived behavioural control, and subjective norms are significantly and positively associated with EFL learners’ adoption intention of mobile English learning, which is in line with the hypotheses of TPB (Ajzen, 1985). Specifically, subjective norms had the strongest influence among the three antecedents, followed by perceived behavioural control and attitude. However, this finding is inconsistent with the previous studies, which suggested no significant association between subjective norms and behavioural intention (Yuen and Ma, 2008; Knauder and Koschmieder, 2019). Excepting the differences in the measurement tools used in the studies, the possible explanation is the age characteristic of the respondents. Respondents in the abovementioned studies were mainly adults older than 25, while the respondents of this study were primarily university students younger than 24. Since the life experiences of students are insufficient, their behavioural intention may be more easily influenced by the opinions of other important people. To better understand the association between subjective norms and behavioural intention, further research can focus on younger students.

### The influence of technostress is not significant

The unexpected finding is that, in the context of mobile English learning, technostress had neither significant influence on adoption intention nor moderating effects in the relationship between subjective norms and adoption intention. This finding contradicts the existing studies suggesting the negative consequences of technostress in work settings (Ayyagari et al., 2011; Tarafdar et al., 2011). It is also inconsistent with the effects of technostress in other learning settings, which suggested that technostress negatively predicts users’ adoption and continuous usage intention of digital textbooks and leads to students’ burnout in technology-enhanced learning (Verkijika, 2019; Zhao et al., 2022). We provide some plausible explanations for this finding: (1) The new generation is considered native to technologies, and most of them have owned smartphones at an early age and have been used smartphones frequently for multiple purposes (van Deursen et al., 2015). Learning English on mobile devices may be just as common as any other smartphone-related activities (e.g., communications, playing video games), which will not make them feel complicated or threatened. (2) Different from the fact that individuals are required to use a given technology in work settings or course learning without choices, in mobile English learning context, to some extent, individuals have more autonomy according to their preferences. (3) As the main activities of mobile English learning are speaking, reading, and listening, the limitations of mobile devices (e.g., limited input mechanism) may not constitute obstacles to learners.

### The important role of compatibility

**FIGURE 4:**
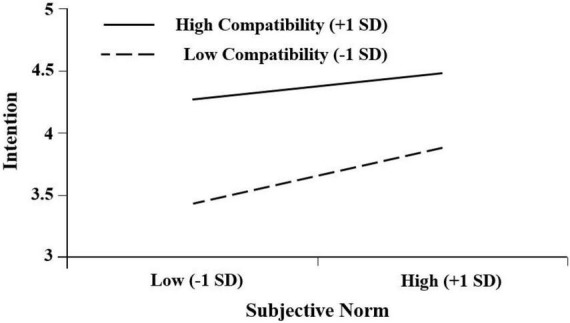
Subjective norms × compatibility for adoption intention.

The results indicated that compatibility significantly predicts intention, suggesting that higher compatibility leads to higher usage intention of mobile English learning. This result is consistent with previous studies which recommended that compatibility significantly predicts behaviour (Wang et al., 2016). However, in this study, compatibility is the strongest precursor of intention. Additionally, it plays a negative moderating role in the effect of subjective norms on adoption intention, indicating that higher compatibility may reduce the impact of subjective norms on intention (Figure 4). This finding suggested that when individuals concern more about the fit of mobile English learning and their learning styles, they will be less concerned about the opinions of important others. It explained the situations in which subjective norms may not influence behavioural intention significantly. All these suggested the predominant role of compatibility in mobile English learning.

### Implications for practice

Our findings have some practical implications related to mobile English learning. First, the opinions of important others on whether they should engage in mobile English learning are important to individuals’ adoption intention. Although who are the important others is still a question, adding check-in services to learning applications that allow users to share their learning experience with wider social circles may help to create a positive impression of mobile English learning in more people’s minds. Second, the finding highlights the critical impact of compatibility, which is the strongest predictor of adoption intention.

Furthermore, when mobile English learning is not compatible with an individual’s learning style and life experience, the effect of subjective norms would be weakened. Therefore, developers need to create an impression that the mobile English learning applications are compatible with their potential adopters’ learning styles. To this end, developers should pay special attention to the factors that may improve users’ compatibility perception, such as perceived performance and entertainment (Jaklič et al., 2018; Jimenez et al., 2019). For instance, the vocabulary learning applications need to filter and provide the vocabulary that the targeted learners need most instead of all-encompassing to help them realise the expected achievements, adding more artificial intelligence teaching aids related to vocabulary comprehension to enhance learners’ embodied and entertainment experience.

### Limitations and future work

There are still some limitations in this study. Firstly, the construct of compatibility was tested with only two valid items. Secondly, the participants came from only one university in China, limiting the results’ generality, as students’ characteristics may differ across regions and countries. Thirdly, as the data were collected from self-reported questionnaires, there may be reporting bias in this study. Future studies collecting data from multiple sources will help further understand EFL learners’ mobile learning adoption. Finally, we only examined the moderating effect of boundary conditions on the influence of subjective norms on behavioural intention. As the influence of attitude on behavioural intention is also inconsistent (Ndibalema, 2014), future research should explore the moderating effect on this path to understand users’ technology adoption better.

## Conclusion

With the lens of the TPB, this study tried to understand the mechanism of EFL learners’ mobile English learning adoption. The results revealed that users’ attitude toward mobile English learning, perceived behavioural control, and subjective norms are positively associated with their adoption intention, and their adoption intention further positively predicts learners’ learning frequency and duration. The results also confirmed the strong effect of compatibility on adoption intention and the negative moderating effect on the influence of subjective norms on intention. However, the moderating role of technostress in this relationship is not found. These findings contribute to the area of mobile English learning from several perspectives. The first is that it extends the existing literature on intention-based research by emphasising the TPB in conjunction with the influences of compatibility and technostress. The nuanced findings provide a plausible explanation for the previous inconsistent results on the relationship between subjective norms and behavioural intention. The second contribution comes from the evaluation of the influence of technostress. As far as we know, this is the original research to examine the effect of technostress on mobile English learning. Although its direct and moderating effects are both insignificant, the finding proves the existence of the situation where technostress does not influence technology adoption, reminding researchers that the results of technostressmay differ across settings. The last contribution comes from the investigation of the impact of compatibility, which suggests the predominant role of compatibility in mobile English learning as the strongest predictor for behavioural intention and a significant negative boundary condition to understand the influence of subjective norms on behavioural intention.

## Data availability statement

The raw data supporting the conclusions of this article will be made available by the authors, without undue reservation.

## Ethics statement

Ethical review and approval was not required for the study on human participants in accordance with the local legislation and institutional requirements. The patients/participants provided their written informed consent to participate in this study.

## Author contributions

QW and GZ completed the manuscript together. ZC completed the data analysis and designed the figures and tables in this study. All authors contributed to the article and approved the submitted version.

## Footnotes

1
https://www.sohu.com/a/415687492_120205287
